# *“Everywhere you see*,* C-sections are happening”*: examining trends and reasons for a rise in cesarean-section deliveries among women in urban informal settlements, Mumbai Metropolitan Region, using a mixed methods approach

**DOI:** 10.1186/s12884-026-09160-8

**Published:** 2026-05-04

**Authors:** Jennifer Spencer, Sheetal Rajan, Rijuta Sawant, Sarita Patil, Shanti Pantvaidya, Vanessa D’Souza, Sushmita Das

**Affiliations:** Society for Nutrition, Education and Health Action (SNEHA), Mumbai, India

**Keywords:** Cesarean section, C-section, Mixed methods research, Urban informal settlements, India

## Abstract

**Background:**

Cesarean sections (C-sections) are life-saving surgeries; however, rates exceeding 10–15% are not associated with reductions in maternal and neonatal mortality. In urban India, C-section rates were 32% in 2019-21. Research on C-section deliveries in urban informal settlements and among those primarily accessing public healthcare services has been limited. This study examined trends and underlying reasons for C-sections in informal settlements in the Mumbai Metropolitan Region, India, drawing insights from both women and obstetric practitioners (public and private). Through this, the study aimed to identify potential suggestions to improve obstetric care in vulnerable settings.

**Methods:**

The study employed an explanatory sequential mixed methods design. Quantitative data were collected through cross-sectional surveys in 2019, 2021, 2022 and 2024, including a pooled sample of 5,162 mothers of children aged 0–2 years. Logistic regression analysis was conducted to identify factors associated with C-sections. Qualitative data collection from October 2024 to February 2025 involved in-depth interviews (*N* = 44) with women who underwent C-sections and public and private practitioners. Thematic analysis was performed using substantive coding to inductively develop themes.

**Results:**

C-section rates increased from 25% in 2019 to 37% in 2024, with a 15% increase in private facilities and 10% increase in public facilities. Maternal age, gravida, timing of antenatal care registration and number of antenatal visits were significantly associated with C-sections. Qualitative findings identified several interconnected reasons, such as institutional limitations, evolving obstetric practices, risk perceptions, awareness and lifestyle changes, to be contributing to C-sections. Likelihood of reported reasons was greater among primigravida women, contributing to a rise in repeat C-sections. Reported reasons were similar between public and private facilities, whereas practitioners and women had divergent perspectives towards rising C-section rates.

**Conclusions:**

C-sections are rising in urban informal settlements among women primarily accessing public facilities. Non-medical reasons influence medical indications and decisions for C-sections. A multi-pronged approach involving both community-based and systemic interventions is needed to reduce avoidable C-sections. This study provides critical evidence to inform interventions for improving patient-practitioner relations and delivery experiences while bettering obstetric outcomes in vulnerable settings.

**Supplementary Information:**

The online version contains supplementary material available at 10.1186/s12884-026-09160-8.

## Background

Cesarean sections (C-sections) are life-saving surgical procedures in situations where vaginal deliveries pose a high risk to pregnant women. Conversely, C-sections can have adverse consequences such as a higher risk of infection and post-natal complications [1, 2]. C-sections are known to reduce maternal mortality, but studies have shown such causations to not hold true when the overall rate of C-section is above 10% [3]. The World Health Organization (WHO) also considers that a C-section rate higher than 10–15% of total deliveries is less associated with reducing maternal and neonatal mortality outcomes [4]. However, over the years, C-section rates have been on the rise, globally accounting for 21% of total deliveries in 2021, up from 7% in 1990 [5]. A rise above 15% has been reported in different regions across the world- 40.5% in the Caribbean, 32.2% in North America, 25% in Europe and 19.2% in Asia [6].

In India, the C-section rates have risen from 8.5% in 2005-06 to 21.5% in 2019-21 according to the National Family Health Surveys (NFHS) conducted by the Government of India [7, 8]. The latest NFHS-5 survey of 2019-21 shows that the proportion of C-section deliveries was much higher in urban areas (32.3%), almost double that of rural areas (17.6%). 49.3% of deliveries in private health facilities were through C-section, while the proportion of C-section deliveries was 22.7% in public health facilities [8].

The decision to undertake a C-section surgery is generally associated with medical indications. These include emergency C-sections due to complications such as fetal distress, breech position, placenta previa, and preterm birth [9, 10]. Other prior-identified medical reasons leading to planned C-section surgeries include twin pregnancies, women with short stature, obesity, prior C-section deliveries and patients with comorbidities [11, 12].

Reasons for a rise in C-section deliveries have been attributed to various factors. Demographic changes, including pregnancies among older women, higher education, higher incomes and awareness of women have been identified with high C-section rates [13, 14]. The rise of C-section deliveries has also been attributed to commercial interests of private facilities [15, 16] and institutional limitations in health facilities [12, 17–19]. Several studies have shown how perceptions of pregnant women, their families and obstetric health staff influence the decision of having C-section deliveries [17, 20–23].

Research related to C-section deliveries among women in urban informal settlements and the reasons thereof has, however, been limited [24–27]. It is important to note that a large proportion of the urban population in low- and middle-income countries (LMICs) lives in inadequate housing and experiences disparities in terms of basic services and healthcare [28]. Women in these vulnerable environments often find themselves caught in a cycle of poor health exacerbated by childbirth and limited access to quality obstetric care, which can lead to complications during pregnancy and delivery [29]. The majority of residents in urban informal settlements seek healthcare through public facilities, where they often encounter limitations of service availability, accessibility, and potential discrimination [30].

Few studies have focused on the issues of poor awareness and agency among women in low-income settings [11, 21, 31]. However, the reasons for rising C-section deliveries among women in vulnerable urban settings, who access mainly public health services, have been less explored. Further, most qualitative studies have explored experiences and perceptions of C-Sections [32–34], whereas reasons have been studied mainly quantitatively [25, 26]. Few studies have explored both practitioner and patient perspectives regarding C-sections and the reasons thereof [11, 20, 35].

In this context, our study aims to holistically analyze C-section trends and the reasons for C-section deliveries in urban informal settlements in one municipal corporation in the Mumbai Metropolitan Region (MMR), India. The MMR is one of India’s largest urban agglomerations; the municipality under study has a population of more than 20 million with 42% living in urban informal settlements [36]. With women in such settlements primarily accessing public health services [30], and one third of all deliveries being C-Sections [37], it is relevant to explore the trends and reasons for C-sections in this prominent urban geography.

Through a mixed methods approach, the study explores trends on C-sections and reasons for C-sections from both women who had undergone C-sections, and practitioners providing obstetrics and gynecology services in public and private health facilities. By doing so, the study aims to identify potential suggestions and interventions to improve obstetric care in vulnerable settings.

## Methodology

### Study design

This is a mixed methods research using the explanatory sequential mixed methods design [38]. Quantitative data was first collected and analyzed, followed by qualitative research. Such a mixed methods design provides the opportunity to analyze and address complex health policy concerns by both mapping overarching trends as well as developing an in-depth understanding of issues.

### Study setting

The study was conducted in a municipal corporation of the MMR. The quantitative surveys (with women participants) were conducted in six urban informal settlement clusters. Among these, the qualitative study (with women participants and private practitioners) was conducted in three urban informal settlements, representative of the quantitative sample areas. Given the nature of maternity referrals and patient choices, women in our study setting accessed public facilities across the municipal corporation; therefore, public facilities included in the study extend beyond these settlements.

The settlements studied are part of the program intervention areas of the Society for Nutrition, Education and Health Action (SNEHA), a non-governmental organization working in urban informal settlements in MMR since 1999. SNEHA engages in various community-level programs to improve maternal and child health and nutrition (MCHN), and works in partnership with public health systems and healthcare providers to enhance the delivery of MCHN services for the urban poor.

Urban informal settlements commonly referred to as slums are characterized by a high population density, inadequate housing, informal and insecure livelihoods, and poor access to basic services [30]. Being a dense urban area, several public and private health facilities were available to the population studied. Public healthcare in the city and within the areas studied is mainly provided by the municipal corporation through primary, secondary and tertiary-level facilities. In the case of maternity care, these range from maternity homes (primary facilities), peripheral hospitals (secondary facilities) and tertiary hospitals and medical colleges (tertiary facilities). Some other facilities managed by the state government are mostly secondary or tertiary-level hospitals. Private facilities in the areas studied ranged from maternity clinics and homes to super-specialty hospitals. A high rate of institutional deliveries (average 98%) was reported in the districts of the areas studied [8].

### Quantitative data collection and analysis

The quantitative component of this study aimed to present trends in C-sections and to analyze factors associated with C-sections. This analysis utilized a pooled subset of data collected through routine evaluation surveys conducted by the Monitoring and Evaluation domain at SNEHA, which operates under an independent framework for monitoring, assessment and reporting of program outcomes and impact.

For this study, data from cross-sectional quantitative surveys conducted in 2019, 2021, 2022, and 2024 were included, comprising a total analytic sample of 5,162 mothers of children aged 0–2 years across survey rounds. As this analysis draws on pooled secondary data from multiple programmatic evaluation surveys, sample sizes for individual rounds were determined by program monitoring requirements rather than a single a priori calculation. These surveys were conducted across six urban informal settlement clusters that served as implementation areas of SNEHA’s MCHN program and included baseline, midline, and endline assessments.

Stratified random sampling was used within each survey round, with Anganwadi catchment areas (government-supported childcare centers in India providing health and nutrition services) serving as strata. A random sample was drawn from a pre-existing line list of households with children aged 0–2 years. This sample was shared with interviewers who conducted household visits to administer the survey. Mothers were eligible to participate if they had resided in the area during the preceding six months and provided informed consent. If a selected target respondent was unavailable after three attempts, the next household in the sample list was approached to maintain survey coverage.

Data collection was conducted through in-person interviews with mothers, each lasting approximately 25–30 minutes. A team of 12 interviewers, supervised by two field officers, was responsible for data collection. Interviewers underwent extensive training on obtaining informed consent, conducting interviews and electronic data collection. Data was collected using CommCare (Dimagi, USA), an open-source, mobile-based platform with a cloud-based server. The interview tool, developed in Hindi and adapted from the NFHS questionnaire [8] captured several maternal and child health indicators. For this study, we extensively utilized data on maternal characteristics (parity), antenatal care (ANC) (month of registration, number of visits), mode of delivery (vaginal/C-section), type of delivery facility (private/public), as well as sociodemographic and household characteristics. The detailed questionnaire can be found in Annexure 1.

Robust data quality controls were employed to minimize errors during collection and entry. The database management system included built-in skip patterns, acceptable ranges, and constraints. Field officers ensured quality through spot checks and back checks. Following on-site digital data collection using tablets, the data were electronically transferred to the CommCare server. Data analysis was performed using STATA (v.14.2) and was periodically checked for completeness and accuracy. Descriptive statistics were used to summarize the demographic, household and service utilization characteristics of the study population. Bivariate analysis was performed using Chi-square tests to examine associations between each covariate and C-section delivery. Additionally, logistic regression was conducted to identify factors associated with C-section deliveries, with covariates including demographic characteristics (age, education, occupation, religion, gravida and duration of residence), household characteristics (family type and socioeconomic status), and service utilization variables (trimester of ANC registration, number of ANC visits and type of delivery facility). In all analyses, statistical significance was set at p-value < 0.05 and odds ratios with 95% confidence intervals (CI) were used to assess the strength of association.

### Qualitative data collection and analysis

The qualitative research primarily aimed to get detailed insight into the reasons for C-sections and perceptions regarding a rise in C-sections in urban informal settlements. Qualitative data collection and analysis was conducted post the analysis of the quantitative data. Qualitative research methods were adopted for an in-depth exploration of practices, perceptions and experiences of women who had undergone C-sections and practitioners providing obstetrics and gynecology services in public and private health facilities.

Women participants were purposively sampled from the most recent (2024) quantitative survey to include those who had a C-section within one year prior to the study, and to ensure the logistic convenience of tracing households. Diversity criteria used for purposive sampling included place of residence, age of the mother, number of prior deliveries and the type of facility where the C-section delivery was done. Practitioners, both public and private, were initially sampled purposively based on responses (names of delivery facilities) from women participants. Thereafter, to ensure a more holistic analysis, convenience sampling was used to identify other public and private facilities around the localities studied. Within each facility, the selection of practitioners depended on their availability and consent to participate.

The field staff in SNEHA, which has strong ties with the community, first identified the households of women participants and approached them for the study. They explained the purpose of the study to women and inquired about their willingness to participate, after which the interviews were accordingly scheduled with those who agreed. Similarly, SNEHA has close ties with the public facilities in the city managed by the municipal corporation. The researchers provided SNEHA’s program staff with the list of public facilities identified through women’s interviews. The program staff accordingly approached public practitioners and explained the purpose of the study. Thereafter, interviews were scheduled with those who were available and agreed to participate. Private practitioners in and around the urban informal settlements were directly identified and approached by the researchers. In all cases, the researchers first explained the purpose of the study, and after a process of informed verbal consent, the interviews were conducted. The exact sample size was not predetermined; information redundancy was used as a criterion to ensure data saturation until which additional participants were identified [39].

The data collection was conducted from October 2024 to February 2025. A total of 44 in-depth interviews were conducted in Hindi and English. Details of the participants can be found in Table 1.

**Table 1:** Participant details of the qualitative study

Participant details	Total
**Participants-Women**	
Total participants	24
Age (years)	
18–24	7
25–29	9
30–34	4
>=35	4
Number of children (living)	
1	8
2	8
3	6
>=4	2
Delivery facility	
Public	14
Private	10
**Participants- Practitioners**	
Total participants	20
Gender	
Male	2
Female	18
Years of experience*	
1 to 10	4
11 to 20	10
> 20	5
Facility	
Public	10
Private	10
Facility level	
Public maternity home	3
Public peripheral hospital	5
Public tertiary hospital	2
Private maternity home	2
Private hospital	8

*Information regarding years of experience was undisclosed by one participant

Authors JS, SR and SD, well-trained in qualitative data collection methods and familiar with the local context, conducted the interviews. Interviews with women were conducted in their homes and lasted for an average of 30 minutes; practitioner interviews were conducted in the health facilities and lasted for an average of 35 minutes. An interview guide was initially prepared based on insights from the quantitative findings and existing literature, and developed iteratively with emerging themes throughout the data collection process. Key points explored in the interview guide can be found in Table 2. Practitioner perceptions and experiences, and women’s pregnancy and delivery experiences were both explored over time. The detailed final interview guide can be found in Annexure 2.

**Table 2:** Key points of the qualitative interview guide

Women who underwent C-sections	• Demographic details • Experience of C-section delivery, events leading up to the delivery • Pre-delivery/Pregnancy details related to awareness and complications • Understanding agency and decision-making • Reasons reported for C-section/s • Post-delivery challenges for maternal and newborn health • Overall perceptions/preferences about C-section and its reasons
Practitioners	• Demographic details • General perspective about the reasons for a rise in C-sections • Exploring reasons for C-sections: medical and non-medical • Regarding antenatal care and post-operative care • Agency in decisions regarding C-sections • Institutional monitoring of C-sections • Experiences/cases of C-section deliveries

Data analysis was performed simultaneously during the data collection period. Interviews of participants who consented to audio-recording were transcribed and translated into English and corroborated by the interviewers. For other interviews, particularly practitioner interviews, detailed notes made during the interview were transcribed into English. Transcripts and detailed notes were analyzed and coded by authors JS and SR using the Taguette Qualitative Analysis Software [40]. Substantive coding, used in grounded analysis [41], which includes open and axial coding, was done to inductively develop emerging themes.

### Ethical considerations

Ethical approval for the quantitative surveys over the years was obtained from the institutional review boards of Sigma Research and Consulting Pvt. Ltd. and Bandra Holy Family Hospital. For the qualitative research, ethical approval was obtained from the MPRS^®^ Research institutional review board. All study procedures were conducted in accordance with the Declaration of Helsinki.

For the quantitative surveys, written informed consent was obtained from the respondents prior to conducting interviews. For the qualitative study, the use of verbal consent was approved by the review board. This was a conscious choice since our qualitative participants, particularly practitioners, are not always comfortable receiving/signing forms due to perceived risk to their profession and institution. Therefore, based on our experience of qualitative studies in urban informal settlements and health systems in the MMR, it was preferred to receive verbal consent for participation. Such consent was audio recorded in cases where participants provided permission for recording. Anonymity of the names of the municipal corporation, hospitals/clinics and participants has been maintained. Interviewers ensured privacy and confidentiality throughout the data collection process. Confidentiality of the information was ensured during data processing, and outputs did not include participants’ names or identifiers.

## Results and findings

### Trends and determinants of rising C-sections: quantitative analysis

A total of 5,162 respondents participated in the surveys (2019, 2021, 2022, 2024). 71% of the respondents were below 30 years of age, and 41.9% had completed secondary education. The majority (90.7%) were unemployed, while 62.6% identified as Muslim. Half of them (50.1%) had three or more children, and 48.4% had been living in the city for more than ten years. More than half of the women lived in nuclear families (54.3%), with a socio-economic status distribution showing 26.7% in the poorest category and 24.2% in the least poor category.

Regarding ANC and maternal health services, 49% registered their pregnancy during the first trimester, and most (94.6%) attended at least four antenatal visits. Additionally, 69.8% delivered in public facilities (Table 3).

**Table 3:** Socio-demographic characteristics of the quantitative study population, stratified by delivery type

Characteristics	Total *N* = 5162		Vaginal *N* = 3495		C-section *N* = 1667	
	*n*	%	*n*	%	*n*	%
**Maternal characteristics**						
Age (years)						
< 20	64	1.2	48	1.4	16	1.0
20–24	1582	30.6	1113	31.8	469	28.1
25–29	2019	39.1	1331	38.1	688	41.3
30–34	1077	20.9	723	20.7	354	21.2
35+	420	8.1	280	8.0	140	8.4
Education						
No schooling	942	18.2	700	20.0	242	14.5
Primary	702	13.6	518	14.8	184	11.0
Secondary	2164	41.9	1435	41.1	729	43.7
Higher	1354	26.2	842	24.1	512	30.7
Occupation						
Not working	4680	90.7	3177	90.9	1503	90.2
Working	482	9.3	318	9.1	164	9.8
Religion						
Muslim	3229	62.6	2243	64.2	986	59.1
Hindu	1811	35.1	1177	33.7	634	38.0
Others	122	2.4	75	2.1	47	2.8
Gravida						
1 pregnancy	1145	22.2	731	20.9	414	24.8
2 pregnancies	1430	27.7	898	25.7	532	31.9
3 or more pregnancies	2587	50.1	1866	53.4	721	43.3
Duration of residence in the city						
< 1 year	63	1.3	52	1.6	11	0.7
1–5 years	1501	30.3	1063	31.9	438	27.2
6–10 years	988	20.0	694	20.8	294	18.3
> 10 years	2395	48.4	1528	45.8	867	53.9
**Household characteristics**						
Family type						
Nuclear	2803	54.3	1956	56.0	847	50.8
Joint	2359	45.7	1539	44.0	820	49.2
Socio-economic status						
Poorest	1285	26.7	916	28.4	369	23.2
Quartile 2	1126	23.4	779	24.1	347	21.8
Quartile 3	1241	25.7	820	25.4	421	26.5
Least poor	1168	24.2	716	22.2	452	28.4
**Service uptake characteristics**						
Trimester of ANC registration						
1st trimester	2530	49.0	1587	45.4	943	56.6
2nd trimester	2073	40.2	1474	42.2	599	35.9
3rd trimester	526	10.2	409	11.7	117	7.0
Not registered	33	0.6	25	0.7	8	0.5
No. of ANC visits						
0–3 ANC visits	277	5.4	226	6.5	51	3.1
4 or more ANC visits	4839	94.6	3232	93.5	1607	96.9
Type of delivery facility						
Public facility	3602	69.8	2471	70.7	1131	67.8
Private facility	1560	30.2	1024	29.3	536	32.2

*Missing data reported in duration of residence in the city (*n* = 215), socio-economic status (*n* = 342) and no. of ANC visits (*n* = 46)

**Fig. 1:**
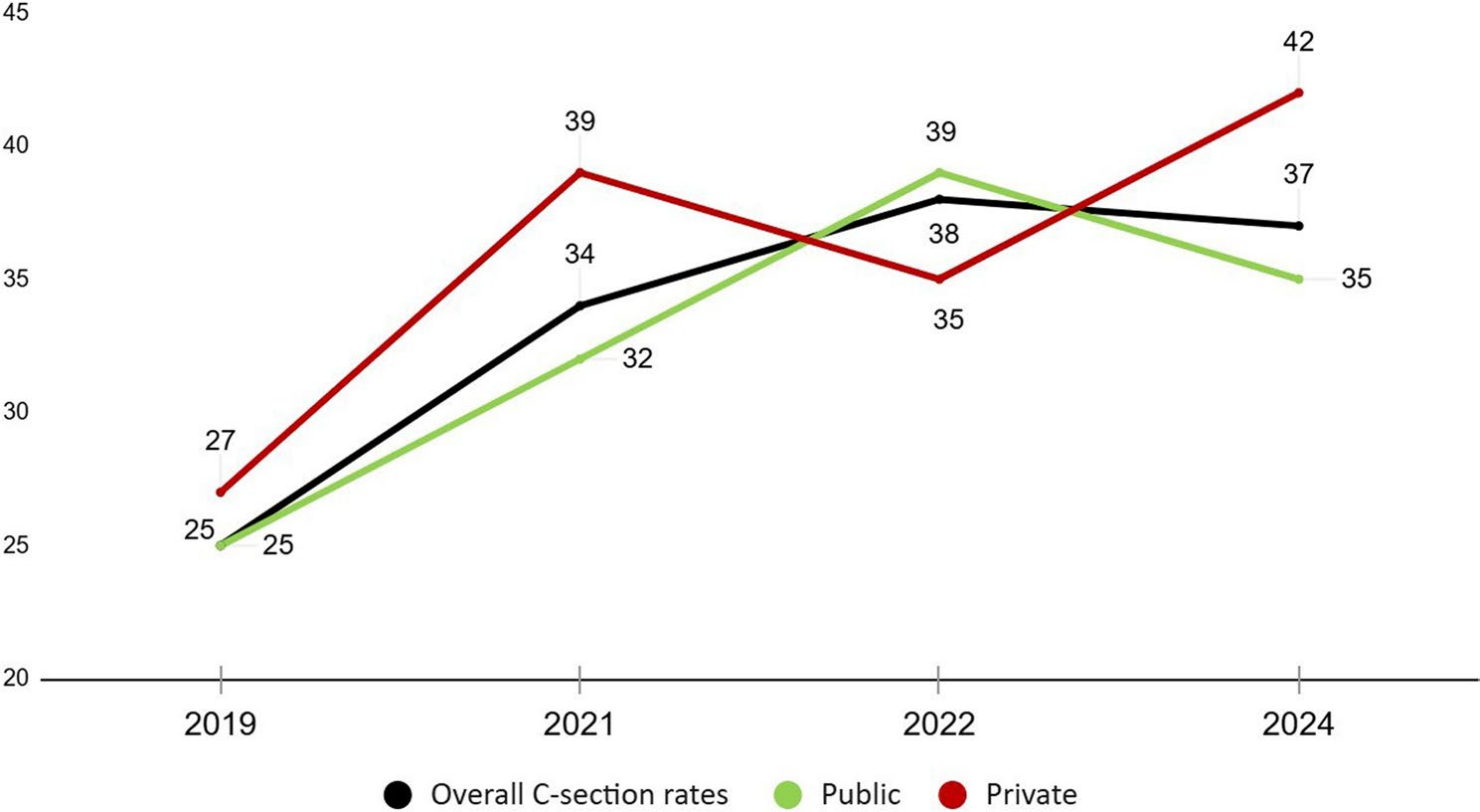
C-section rates in public and private facilities among women in urban informal settlements, MMR (2019-24)

Our quantitative data (Fig. 1) shows that overall C-section deliveries have been rising in the urban informal settlements studied, from 25% in 2019 to 37% in 2024, where private facilities have seen a percentage increase of 15% and public facilities of 10% in the intervening years. Notably, as seen in Fig. 1, there was a substantial rise in C-sections from 2019 to 2021, coinciding with the period of the coronavirus disease (COVID-19) pandemic.

While the rate of C-sections to total deliveries was higher in private facilities, the proportion of total C-section deliveries by facility shows that in our setting, almost two-thirds of the C-sections were taking place in public facilities (Table 3). This shows that most patients from informal settlements used public facilities for obstetric care, pointing to the need for further analysis of rising C-sections among those accessing public facilities.

The analysis examined factors associated with C-section deliveries among women, using both bivariate and multivariate logistic regression models. Women aged 35 years and older had higher odds of C-section compared to those under 20 years in adjusted models (AOR: 2.55, 95% CI: 1.32–4.96). Women with three or more pregnancies had significantly reduced odds of C-section (AOR: 0.64, 95% CI: 0.53–0.78) compared to primigravida women. Duration of residence in the city also influenced outcomes, with women residing for over 10 years showing increased odds of C-section delivery (AOR: 2.00, 95% CI: 1.01–3.93). ANC characteristics played a critical role. Later ANC registration in the second and third trimesters was associated with lower C-section likelihood (AOR: 0.81 and 0.67, respectively), while a higher frequency of ANC visits increased the odds of C-sections (AOR: 1.71, 95% CI: 1.22–2.39). Details are presented in Table 4.

**Table 4:** Unadjusted and adjusted logistic regression models for the determinants of C-section deliveries

Variables	Bivariate		Adjusted	
	OR	95% CI	AOR	95% CI
**Maternal characteristics**				
Age (years)				
< 20			1	[1,1]
20–24	1.30	[0.73–2.31]	1.51	[0.81–2.80]
25–29	1.57	[0.88–2.79]	2.11*	[1.13–3.94]
30–34	1.47	[0.82–2.63]	2.19*	[ 1.16–4.14]
35+	1.56	[0.85–2.85]	2.55**	[1.32–4.96]
Education				
No schooling			1.00	[1,1]
Primary	1.01	[0.81–1.27]	0.98	[0.77–1.24]
Secondary	1.40***	[ 1.17–1.66]	1.22*	[1.01–1.49]
Higher	1.59***	[ 1.32–1.92]	1.22	[0.98–1.53]
Occupation				
Not working			1	[1,1]
Working	1.09	[0.89- 1.33]	1.09	[0.89–1.35]
Religion				
Muslim			1	[1,1]
Hindu	1.09	[0.96-1.25]	0.99	[0.86–1.15]
Others	1.30	[0.89–1.89]	1.08	[0.73–1.60]
Gravida				
1 pregnancy			1	[1,1]
2 pregnancies	1.04	[0.88-1.22]	1.03	[0.86–1.22]
3 or more pregnancies	0.70***	[0.60–0.81]	0.64***	[0.53–0.78]
Duration of residence in the city				
< 1 year			1	[1,1]
1–5 years	1.83	[0.94–3.56]	1.62	[0.82–3.17]
6–10 years	1.86	[0.95–3.63]	1.72	[0.87–3.40]
> 10 years	2.52**	[1.30–4.88]	2.00*	[1.01–3.93]
**Household characteristics**				
Family type				
Joint			1	[1,1]
Nuclear	0.83**	[0.74–0.94]	0.95	[0.83–1.09]
Socio-economic status				
Poorest			1	[1,1]
Quartile 2	1.10	[0.93- 1.32]	1.00	[0.84–1.20]
Quartile 3	1.24	[1.04–1.47]	1.03	[0.85–1.23]
Least poor	1.54***	[1.29–1.82]	1.16	[0.95–1.41]
** Service uptake characteristics**				
Trimester of ANC registration				
1st trimester			1	[1,1]
2nd trimester	0.71***	[0.62–0.80]	0.81**	[0.71–0.93]
3rd trimester	0.49***	[0.39–0.61]	0.67**	[0.53–0.86]
No. of ANC visits				
0–3 ANC visits			1	[1,1]
4 or more ANC visits	2.22***	[1.62–3.01]	1.71**	[1.22–2.39]
Type of delivery facility				
Public facility			1	[1,1]
Private facility	1.14*	[1.00-1.30]	1.13	[0.98–1.29]

*p value < 0.05, ** *p* value < 0.01, *** *p* value < 0.001

AOR adjusted for all variables listed in the table

*OR:* Odds Ratio, *AOR:* Adjusted Odds Ratio, *CI:* Confidence Interval

### Narratives of women and practitioners on reasons for C-section: qualitative insights

Based on the in-depth interviews with women who had undergone C-sections and practitioners providing obstetrics and gynecology services in public and private facilities, we inductively developed themes and sub-themes elucidating women and practitioner perspectives and experiences about the reasons for C-sections.

#### Medical and clinical indications

C-sections were reported to have been conducted in indicative cases only, for various maternal and fetal conditions that necessitated surgical intervention. Maternal conditions driving C-sections included short stature, placenta previa, maternal fits, prior illnesses like asthma, obesity, pregnancy-induced hypertension (PIH) and gestational diabetes. Clinicians reported that while existing risks of malnutrition, like anemia among women persist, there was an emerging trend of non-communicable diseases (NCDs) that increased the likelihood of a C-section procedure.

“Endocrine disorders in pregnancy are on the rise now, like thyroid, diabetes and other NCDs, and the trend has reversed from earlier risks like malnutrition, anemia and other deficiency disorders. These play with the psyche of the doctor because they increase the risk of stillbirths.” (Practitioner, Public Peripheral Hospital).

Fetal reasons reported were big baby, breech presentation, cord around the neck, fetal heart anomalies, intrauterine growth retardation (IUGR), oligohydramnios and meconium-stained amniotic fluid.

“They said my baby weighed 3.5 kg, and since it was my first delivery, anything could happen. They also said that since the baby was in a breech position, a normal delivery could be risky.” (24-year-old multiparous woman).

Other medical reasons during labor and delivery included non-progress of labor, failed induction, obstructed labor due to cephalo-pelvic disproportion and post-datism. In multiple accounts, the lack of adequate pain, despite the administration of medications intended to induce labor, was interpreted by both women and practitioners as a red flag for non-progress of labor, often leading to the decision for a C-section.

Another reason attributed to rising C-section rates was previous C-sections. Vaginal birth after a previous C-section (VBAC) or trial of labor after a previous C-section (TOLAC) were rarely considered in private hospitals or public maternity homes due to associated risks like uterine rupture, severe postpartum hemorrhage, etc.

“I thought that it had been 8–9 years since the first delivery, so maybe it would be normal (delivery). But when the time came, they said, as you had a cesarean in your first delivery, so you will have to do a cesarean again.” (28-year-old multiparous woman).

Though public tertiary hospitals admitted to giving a trial of labor after assessment of the clinical presentation, they had to seek consent from the patient. When they related the “*0.4-4% risk*” (*Practitioner*,* Public Peripheral Hospital*) associated with a VBAC/TOLAC, most patients opted for a C-section. Thus, the practice of repeat C-sections after a primary C-section was one reason contributing to the rise in overall section rates.

#### Institutional and practitioner-related

Institutional characteristics were reported by both practitioners and women to be one reason shaping C-section decisions.

##### Infrastructure

A lack of availability of medical facilities such as 24 × 7 blood banks and neonatal intensive care units (NICU) were reported to reduce the risk-taking ability of facilities to attempt vaginal deliveries. Private facilities and smaller public facilities, with limited infrastructure, particularly reported facing these challenges. Pregnant women also perceived a lack of available facilities to contribute to C-sections.

“Doctors don’t want to waste time. Some hospitals wait, but others don’t have enough delivery beds, so they don’t wait for a normal delivery. If they wait for a normal delivery, where will they accommodate other patients? That is why they do C-sections.” (34-year-old primiparous woman).

A lack of other facilities, such as a functional operation theatre and medical equipment, along with the above-mentioned limitations, influenced transfers from one hospital to another, at times leading to delays and medical complications necessitating C-sections. In some cases, women also preferred not to be transferred (for a trial of labor to tertiary hospitals) and rather agreed to C-sections.

Although tertiary hospitals reported having facilities, they were severely overburdened due to a high patient load. This led to institutional policies such as NICU facilities reserved only for in-patient deliveries, and no option for labor analgesia, which indirectly contributed to a rise in C-sections.

##### Human resources

Apart from physical infrastructure, most public facilities reported being understaffed; a high patient inflow led the existing staff to be overburdened with cases, often prompting time-saving C-sections. Particularly, the lack of adequate support staff limited the ability to continuously monitor patients during a trial of labor. This was particularly considered a reason for C-section among primigravida women, whose labor and delivery necessitated much more time and monitoring.

“A primi (gravida) woman has to be continuously monitored, but for that, you need proper staff, and in public hospitals, everyone is overburdened. Moreover, doctors don’t have the patience and time to wait for so long in the case of primi deliveries, that is why C-sections are rising. In the public hospital, we still do try for some time, but private hospitals do not want to take much risk, so they wait even lesser.” (Practitioner, Public Peripheral Hospital).

A staff crunch in public hospitals also reflected in poor counseling provided to patients during ANC and postnatal care (PNC) visits.

“Counseling is very important in ANC, however, we have 250–300 patients and only 6 doctors, making it difficult to adequately counsel everyone. General information, such as when a C-section is done and why, its benefits and drawbacks are not given.” (Practitioner, Public Tertiary Hospital).

Doctors felt that they did not have the time to guide patients regarding C-sections and their possibility, complications during pregnancy, family planning practices, or post-section care. This often contributed to patients’ low awareness and led to complications, which culminated in C-section deliveries.

Overburdening of public hospitals also led doctors and staff to become short-tempered or rude with their patients; such experiences, particularly during ANC, generated fear among women to have a vaginal delivery. The overall environment of the public hospitals, where women did not feel cared for, in a few cases, contributed to decisions of elective sections.

“At the *(tertiary)* hospital, they were not caring for people, and there was so much rush there. They told me that a normal delivery is possible, but I will have to wait. I saw other women crying out in pain; they had been waiting for a day or more, and neither their C-section nor normal delivery was happening. Seeing all this, I was scared and feeling nauseated, so eventually I just told the doctor to do a C-section for me.” (35-year-old multiparous woman).

Absence of specialist doctors such as cardiologists, neurologists, etc., who may be required for emergencies, and no 24 × 7 availability of pediatricians or anesthetists, mainly in private hospitals and smaller public facilities, also led facilities to not take the risk of attempting vaginal deliveries with complications.

##### Monitoring

In private facilities, the decision for a C-section was taken by the sole attending doctor, making such decisions more dependent on individual assessment of the risk involved. Moreover, private practitioners reported that institutional monitoring of C-section rates or audit of the C-sections based on medical indication was not done. This contributed to reduced accountability among practitioners for their decisions.

“There are no regulations from the hospital regarding sections, it is solely dependent on the doctor whether to do a normal or C-section delivery. Neither does the facility monitor, nor do I personally keep a record.” (Practitioner, Private Hospital).

Whereas in the case of public facilities, a hierarchy of medical staff and doctors were reported to be involved in each C-section decision, reducing the possibility of a judgment error. However, in public facilities, such institutional hierarchies also meant that senior doctors, not present at the delivery table, remotely directed decisions regarding C-sections.

Monitoring and audit of C-sections in public facilities was also limited to reporting C-section rates; patient-wise audits of medical indications for C-section were not done.

“We have a system of auditing C-sections. However, it is only limited to looking at the section rate. There is no detailed audit involving each indication for every patient.” (Practitioner, Public Tertiary Hospital).

##### Monetary costs

The primary institutional difference between public and private facilities was the monetary costs involved in C-section deliveries. Given the financial constraints faced by women in urban informal settlements, they preferred public facilities due to minimal or no costs. Those who could afford private facilities preferred them due to better care and greater patient-practitioner trust than in public hospitals. However, delivery in private hospitals involved considerable out-of-pocket expenditures.

“In some hospitals, there are scams, like in private hospitals, they do C-sections because it makes them more money. A normal delivery costs ₹20,000, while a C-section costs more than double, around ₹60,000-₹70,000.” (34-year-old primiparous woman).

High costs in private facilities contributed to community perceptions that C-sections were performed for financial gain. Practitioners were often paid per delivery, with higher remuneration for C-sections, unlike public hospitals, where salaries were fixed regardless of delivery type. Nevertheless, most of our practitioners disputed these claims, emphasizing that C-sections were performed based on medical indications and not financial incentives.

“People have the misconception that C-sections are done just like that, or for money, but that is not the case. I don’t think anyone is doing sections purposely. We suggest a section based on indications only.” (Practitioner, Private Hospital).

Private practitioners also highlighted their sense of responsibility towards their patients due to their proximity to the community and trust built over time. To counter negative perceptions surrounding C-sections done for monetary benefit, some private hospitals adopted uniform charges for vaginal and C-section deliveries. Additionally, some private doctors referred high-risk cases to public facilities, where post-delivery care, such as NICU, would be more affordable for patients.

#### Obstetric practices and guidelines

A change in obstetric practices over the years, for instance, advancements in technology such as fetal monitors (Doppler) have increased the ability to pick up complications, which has led to a rise in C-sections. Doctors mentioned that if treatment modalities such as imaging techniques have improved; these should be used. As mentioned by one public practitioner in a peripheral hospital, *“If something is known*,* why shouldn’t the alternative be tried*,* because ultimately a healthy baby should be the priority”.* Practitioners added that early registration of pregnancy has increased over time and subsequently the number of sonographies and follow-ups, leading to better detection of high-risk cases, corroborated through our quantitative analysis (Table 4).

Evolving obstetric guidelines were also reported to have contributed to a rise in C-section deliveries. For instance, practitioners explained how breech position was initially not an indication for operative delivery. However, research showing that planned C-sections yield better outcomes than vaginal births for term fetuses in breech presentation has led practitioners to avoid trial of labor for breech positions.

It was also reported that medical training has changed to favor C-sections. Instrumental deliveries, such as forceps or vacuum are not being practiced, with young doctors lacking training and confidence in these techniques, leading to a preference for C-sections. This preference extends to cases of previous C-sections, where ‘once a C-section, always a C-section’ has become a norm, as doctors are not trained to consider vaginal deliveries in such scenarios.

“I wouldn’t even call it (instrumental delivery) a dying art, rather, it has come to a dead end. It is not being done here, neither are the teachers teaching it, nor do the doctors have the confidence.” (Practitioner, Public Peripheral Hospital).

With evolving obstetric practices, a shift in practitioners’ attitudes towards C-section deliveries was also observed. Senior practitioners reported a generational change, highlighting that younger doctors increasingly prefer C-sections. It was noted that C-sections have become so routine that even residents now undertake cases involving one or two previous cesarean procedures with ease, cases that were traditionally handled by senior doctors.

“This is a tissue paper generation. Young doctors have very little patience, getting a patient to deliver normally is very demanding, but tender loving care is missing in this generation of doctors.” (Practitioner, Public Peripheral Hospital).

Finally, obstetrics as a branch of medicine was regarded as a defensive practice, with neither doctors nor patients willing to take risks.

#### Risk perceptions

The study revealed nuanced risk perceptions among both practitioners and women, which influenced the decision-making process regarding the mode of childbirth. Participants revealed concerns about potential complications, safety and outcomes tied to vaginal birth, which influenced their preference for C-sections.

##### Practitioners’ risk perceptions

Practitioners highlighted that they feel more in control during a C-section, which was perceived to involve lesser risk compared to a vaginal delivery. This perspective was especially prevalent in cases of previous C-sections, where planned surgeries are preferred before the onset of labor. The perception of risk and the associated legal and reputational implications often influenced the preference for C-sections. Doctors expressed concerns that, in the event of negative outcomes, they alone bear the blame.

“Times have changed now; we cannot take a lot of risk - people will come and lynch us. After that, the police will come and ask for video proof, but we will be gone by then. If we take risks, we face public ire, so now no doctor wants to take risks. This is the only reason why there are C-sections – it is not for financial gain but for safety.” (Practitioner, Private Maternity Home).

This risk aversion was more pronounced in private facilities due to limitations such as the absence of NICUs, blood banks, or round-the-clock specialists like anesthetists and pediatricians. Moreover, as earlier mentioned, decision-making in private settings often rested with a single doctor, unlike public hospitals, where delivery decisions were typically made by a team of professionals. As a result, private practitioners frequently screened patients to avoid high-risk cases, referring such cases to public tertiary hospitals.

“In public hospitals, you can take risks because they have a lot of facilities like NICU, blood bank, specialists, so if anything happens to the mother or child, they can provide immediate care; that is not the case with private hospitals.” (Practitioner, Private Hospital).

Practitioners also had a low risk perception of the adverse consequences of C-section. Doctors instead viewed C-sections as a medical advancement aimed at saving lives. They also pointed out that vaginal births carry risks, such as birth injuries, potentially leading to conditions like cerebral palsy (CP). As one public practitioner from a peripheral hospital remarked, *“Just because one can’t afford to go to a private hospital and see a gynecologist of their choice*,* do they have to end up with a child with CP because we in public hospitals want to bring the C-section rate down?”*

Doctors generally perceived that the risk of C-section complications for both mothers and babies was low. For instance, when questioned about increased backaches post C-sections reported by women in our study, doctors attributed these issues to poor nutrition, inadequate supplementation, and improper breastfeeding postures rather than to the surgery itself. While they acknowledged delays in early initiation of breastfeeding– *“the first four hours*,* the golden hours are missed*,* and the first feed isn’t the mother’s” (Practitioner*,* Public Peripheral Hospital)*–this was not regarded as a negative consequence of C-sections.

##### Women’s risk perceptions

Women’s risk perception was primarily associated with the well-being of their baby and was often driven by fear, particularly among primigravida women. Some women felt that their lack of awareness, combined with the authority of doctors, limited their agency, leaving them with no choice but to comply with medical advice. Risk perception was also influenced by doctors’ confidence and assurance, especially in VBAC/TOLAC cases. Women often reported that doctors were unwilling to take responsibility if complications arose during a trial of labor. As one woman participant explained:

“They said they would attempt a normal delivery, but if any complications arose, they would not take responsibility. Since no doctor guaranteed mine or my baby’s safety, I signed the C-section consent form.” (24-year-old multiparous woman).

Staff behavior and institutional environment, as mentioned previously, heightened women’s risk perceptions during delivery.

Previous adverse obstetric experiences or in vitro fertilization (IVF) pregnancies significantly shaped women’s preferences for C-sections. They viewed their babies as precious and felt unable to take any risks in such situations.

“I had my child after almost 17 years. I was not in a position to take any risks that might cause me to lose my child. When the doctor suggested it (C-section), I immediately agreed.” (35-year-old multiparous woman).

#### Social and demographic reasons

Various social and demographic reasons associated with pregnant women and their health and lifestyle affected the possibility of a rise in C-sections in our context.

##### Awareness

Poor education and awareness among women regarding pregnancy and delivery, particularly among primigravida women, was reported as a reason for a higher possibility of C-sections. For instance, low awareness of the need to timely address ANC complications, take prescribed supplementation, go for regular check-ups and at their estimated due date, often led to unsolved complications which necessitated C-sections.

“Since I had no pain, why would I go? Also, there was no one at home to take care of me. That’s why I decided to go only when I was in pain. Because I had no pain, I went late, and in that they did a C-section, saying I had delayed it. It was my first child, and I had no knowledge of these things.” (24-year-old multiparous woman).

As seen in the quote above, coupled with poor awareness, a lack of family support during pregnancy was seen to aggravate the possibility of complications leading to C-sections.

##### Family planning

Practitioners mentioned that as per obstetric guidelines, an inter-conception period of at least two years is necessary if a vaginal delivery has to be attempted post a C-section. However, a lack of awareness regarding spacing between children and poor family planning behaviors led to repeat C-sections.

“Increasing sections could be attributed to very poor family planning practices with a short inter-conception period, where VBAC cannot be attempted. We are able to give family planning advice only during labor and at the time of discharge. Women don’t come for PNC.” (Practitioner, Public Tertiary Hospital).

Practitioners also felt that stigma and religious beliefs surrounding sterilization led to poor uptake of family planning methods. However, some women felt that to meet family planning targets, public hospitals did C-sections for multiparous women in order to perform tubal ligation along with the delivery.

##### Nutrition and lifestyle

Contemporary lifestyle changes involving food intake and nutrition, sedentary behavior and posture, excessive screen time, erratic sleep patterns, etc., were reported by women and practitioners to have increased complications during pregnancies, leading to C-sections. This was particularly associated with young women, increasing the chances of C-section in primigravida cases.

“C-sections are rising because of the sedentary lifestyle of women with disrupted routines. They also don’t have a healthy diet. Therefore, obstetric outcomes aren’t good, and PIH, IUGR, oligohydramnios have become very common. This is an effect of habits since adolescence. I see 15–16 year-old girls not conscious about their diet and exercise, spending long hours on the phone, having gynecological issues.” (Practitioner, Private Hospital).

Lifestyle changes were also reported to increase infertility and IVF pregnancies that were more likely to result in C-sections. As mentioned by one practitioner:

“People take so much effort in doing an IVF, and after that, both women and doctors don’t want to take a chance. Also, in many cases of IVF pregnancy, there are twins, or the baby has to be delivered preterm, necessitating C-section.” (Practitioner, Public Tertiary Hospital).

#### Context-specific issues

Apart from socio-demographic reasons, certain conditions specific to the context of urban informal settlements in the areas and time period studied also influenced the likelihood of C-section deliveries.

First, as seen in the quantitative analysis, there was an overall rise in C-section rates during the COVID-19 pandemic in the urban informal settlements studied. However, most of our participants did not associate COVID-19 with a rise in C-sections. Few mentioned that relaxations in indications for C-sections were made primarily due to institutional limitations during the pandemic.

“We had more liberal indications for C-sections, our threshold was lower. For instance, for those with meconium, an immediate section was done, a trial was not given.” (Practitioner, Public Peripheral Hospital).

Women participants mentioned that the COVID-19 pandemic influenced their choice of delivery facility– many public hospitals were dedicated to COVID-19 patients, due to which pregnant women chose private hospitals for delivery. While some women attributed delivery in private facilities to C-sections, this was not corroborated by private practitioners.

The specific context of urban informal settlements also influenced C-sections. Poor sanitation and hygiene due to lack of proper waste management and sewage facilities, cramped housing and pollution led to high rates of infections. Infections among pregnant women were particularly related to complications leading to C-section deliveries.

“The infections in the area are very high, leading to pelvic inflammatory disease. Some patients have genital infections with severe white discharge. So, in such cases, C-sections are more likely.” (Practitioner, Private hospital).

Another context-specific factor found in one of the urban informal settlements was associated with a high rate of fetal anomalies. These were related to proximity to a waste dumping ground, the practice of consanguineous marriages among a religious community, delay in anomaly scans, and taboo of abortions. Consequently, such cases of fetal anomalies were delivered through C-sections due to the complications involved.

### Perceptions of practitioners and women towards a rise in C-section rates

We have examined the reasons for C-sections using both practitioner and women perspectives. Building on these findings, we now explore participants’ perceptions of rising C-section rates. Interestingly, there is a clear divergence in how practitioners and women interpret the rise in surgical births, reflecting distinct concerns and priorities.

#### Practitioners’ perceptions towards a rise in C-sections

While most practitioners (17 out of 20) acknowledged that C-sections were rising, they didn’t view the trend as an issue warranting close monitoring. Instead, their primary focus was on balancing maternal and neonatal safety with institutional limitations, often prioritizing immediate clinical outcomes over broader efforts to reduce C-section rates.

“I don’t care if anybody says to me ‘your section rate is high’. I only care about my patient.” (Practitioner, Public Peripheral Hospital).

Additionally, many practitioners expressed skepticism about the applicability of global guidelines, such as the WHO-recommended C-section rate of 10–15% [4]. They argued that rigid benchmarks failed to account for local healthcare realities, institutional constraints and country-specific maternal health challenges.

“Fixing a range like 15–20% by WHO is wrong. When you prioritize a safe mother and child, you cannot see the rate. Also, the guidelines cannot be universalized, context differs from country to country. In fact, even within the municipality, there cannot be blanket rates”. (Practitioner, Public Tertiary Hospital)

In the public sector, variations in facility-level infrastructure further complicated assessments of C-section rates.

“One must not assess the C-section rate of maternity homes, rather, do it for medical colleges where all facilities are available. We are trying to do the best we can with limited resources.” (Practitioner, Public Maternity Home).

“We cannot assess our hospital’s C-section rates, since most of the admissions are high-risk cases. Since it is a tertiary level hospital, we get a large chunk of C-section referrals, so our data is skewed.” (Practitioner, Public Tertiary Hospital).

As seen in the above quotes, both primary and tertiary facilities had reservations about their C-section rates being assessed.

#### Women’s perceptions towards a rise in C-sections

Women in our sample acknowledged the increasing prevalence of C-sections, yet their attitudes toward this trend were negative. *“Yes*,* C-sections are happening everywhere. At the hospital*,* I saw many C-sections; it seemed like everyone was having one. People say they are increasing*,* even in the village*,*”* shared one participant (*28-year-old multiparous woman*), reflecting broader perceptions of the procedure’s growing commonality. Despite this, most women expressed a strong desire for vaginal birth. For many, undergoing a C-section was not merely a medical procedure but an experience with lasting repercussions that shaped their physical health, daily functioning and social standing.

Several women reported adverse health consequences following a C-section delivery, citing persistent backaches, digestive issues, negative body image and limitations in physical movement. The inability to lift heavy objects, climb stairs or engage in strenuous household tasks was a recurring concern, leading many to feel restricted in their daily lives.

“I was hoping it would be a normal delivery. Because after an operation, the body becomes useless. Neither can I lift things easily, nor can I do any work properly.” (28-year-old multiparous woman).

Beyond physical challenges, C-section births also carried social and familial implications. Some women faced scrutiny and reproach from family members, particularly from in-laws, who viewed their limited ability to perform household duties as a burden. This strained family dynamics and, in some cases, resulted in emotional distress.

“I told them (the medical staff) not to do the operation, my in-laws will make life difficult for me. And today, my in-laws are taunting me, saying you can’t work. They look for excuses to send me away.” (25-year-old primiparous woman).

Additionally, women expressed concerns that C-section births limited their reproductive choices. Many believed that undergoing C-sections placed restrictions on the number of children they could have, shaping their long-term family planning decisions.

Rise in C-section rates, not a concern for practitioners and considered ‘life-saving’, were thus stigmatized and regarded as ‘life-altering’ surgeries in the community. Table 5 summarizes some of the positive and adverse perceptions of practitioners and women towards C-section deliveries. These contrasting perspectives highlight the complexities surrounding the increasing prevalence of C-sections.

**Table 5:** Perceived benefits and adverse effects of C-sections

Perceived benefits/positive effects of C-sections	Perceived adverse/negative effects of C-sections
• C-section reduces maternal and neonatal mortality risk • It minimizes medico-legal risk for practitioners • It is considered safer in case of precious baby (IVF pregnancies) • Considered a time saving and safer option for delivery in resource-constrained settings	• C-section leads to health complications for women and limits their future reproductive choices • It leads to stigma and strained family relations for women • C-sections increase chances of poor early breastfeeding practices • Practitioners’ preference for C-sections reduces possibility of alternatives (like instrumental delivery)

## Discussion

Our paper aimed at holistically interpreting trends and reasons for a rise in C-sections in urban informal settlements in the MMR through a mixed methods study. The study used quantitative data spanning four years to explain rising trends of C-section deliveries in vulnerable urban settlements. Through the follow-up qualitative research, the study explored both women and practitioner perspectives to inductively develop themes explaining the key reasons for C-section deliveries in these settlements.

Our study shows that C-sections are rising in vulnerable urban settlements. While rising C-section rates have long been attributed to private facilities [15, 16], women in these settlements who underwent C-sections primarily accessed public facilities. Although private facilities also showed higher rates of C-sections to total deliveries, the difference was not as stark as reported in national surveys [8]. Our qualitative data also corroborates that the monetary benefits of private facilities were not a primary reason attributed to rising C-sections. High section rates among those accessing public facilities then points to the need to look beyond the public-private dichotomy [3, 16] in analyzing a rise in C-sections. Based on qualitative insights from practitioners, we can infer that while the reported reasons for C-sections were similar for both public and private facilities, their degree or extent varied. For instance, private hospitals in our context, which were small facilities in the vicinity of urban informal settlements, faced infrastructure limitations due to which practitioners preferred taking lesser risks. This was similar to public maternity homes, but differed from tertiary public facilities willing to take more risk. Within public facilities as well, both maternity homes and tertiary hospitals mentioned limitations due to which they did not prefer their C-section rates to be assessed and rather passed the responsibility to one another. Moreover, as seen through the quantitative data, health crises such as the COVID-19 pandemic led to a stark rise in C-sections, reflecting the immense pressure faced due to institutional limitations in both public and private facilities. This was also seen in other studies done during the pandemic [42, 43]. We therefore argue that a rise in C-sections cannot be based only on public/private or small/big categories of health facilities and rather needs to be situated in the context of where and how each facility operates and whom it serves. The reasons reported, affecting the various facilities in our context, thus need to be collectively addressed for improving obstetric outcomes.

While we recognize the need for addressing rising sections across facilities, in the case of women, our study found gravida status or number of prior deliveries to be a primary demographic influence contributing to rising sections. Prior literature on the subject reports several demographic characteristics associated with C-Sections [13, 14], some of which, like age and ANC uptake, were also reported through our quantitative analysis. However, both our quantitative analysis and qualitative insights show that the likelihood of C-sections was higher among primigravida women. Several of the reasons reported–be it institutional limitations, poor awareness or lifestyle changes–affected primigravida women more. Due to the practice of ‘once a C-section, always a C-section’, repeat sections of such primiparous women contributed to rising rates. Few other studies have also related C-sections to primigravida women; however, these were mainly attributed to patient fear and risk perceptions [20, 44]. This points to the need for ameliorating reasons for sections among primigravida women to address the rise in C-sections.

Our study identified various reasons for C-section deliveries, derived through inductively analyzed themes. These reasons did not operate in isolation; rather, they were interdependent and collectively influenced C-section decisions. For instance, institutional limitations such as understaffing and infrastructure constraints translated into high risk perceptions and overburdening among practitioners. This in turn contributed to poor counseling and low awareness among women, already disadvantaged due to their context-specificities, accentuating medical complications and ultimately leading to C-sections. Throughout the patient journey from the time of ANC to post-delivery, a key insight running across these interconnected reasons was that of poor patient-practitioner interactions. As seen through our findings, practitioner perspectives and motivations towards C-sections differed greatly from those of women, which led to poor communication and sharing of information and concerns among them. A fractured patient-practitioner relationship, as seen in other LMIC contexts as well [24, 31, 34], ultimately led to negative C-section experiences, maintaining a stigma surrounding C-sections and contributing to C-section deliveries.

### Recommendations

Our study recognizes the need to adopt a multi-pronged approach to address the interconnected reasons contributing to rising C-sections. Improving patient-practitioner relations, particularly in the public sector, which is most accessed by vulnerable communities, can enhance overall delivery experience, reduce misconceptions and stigma, and in the long term help reduce avoidable C-sections. Through our inductively developed themes, we accordingly identify strategies within each of these, collectively aimed at reducing avoidable C-sections.

**Fig. 2:**
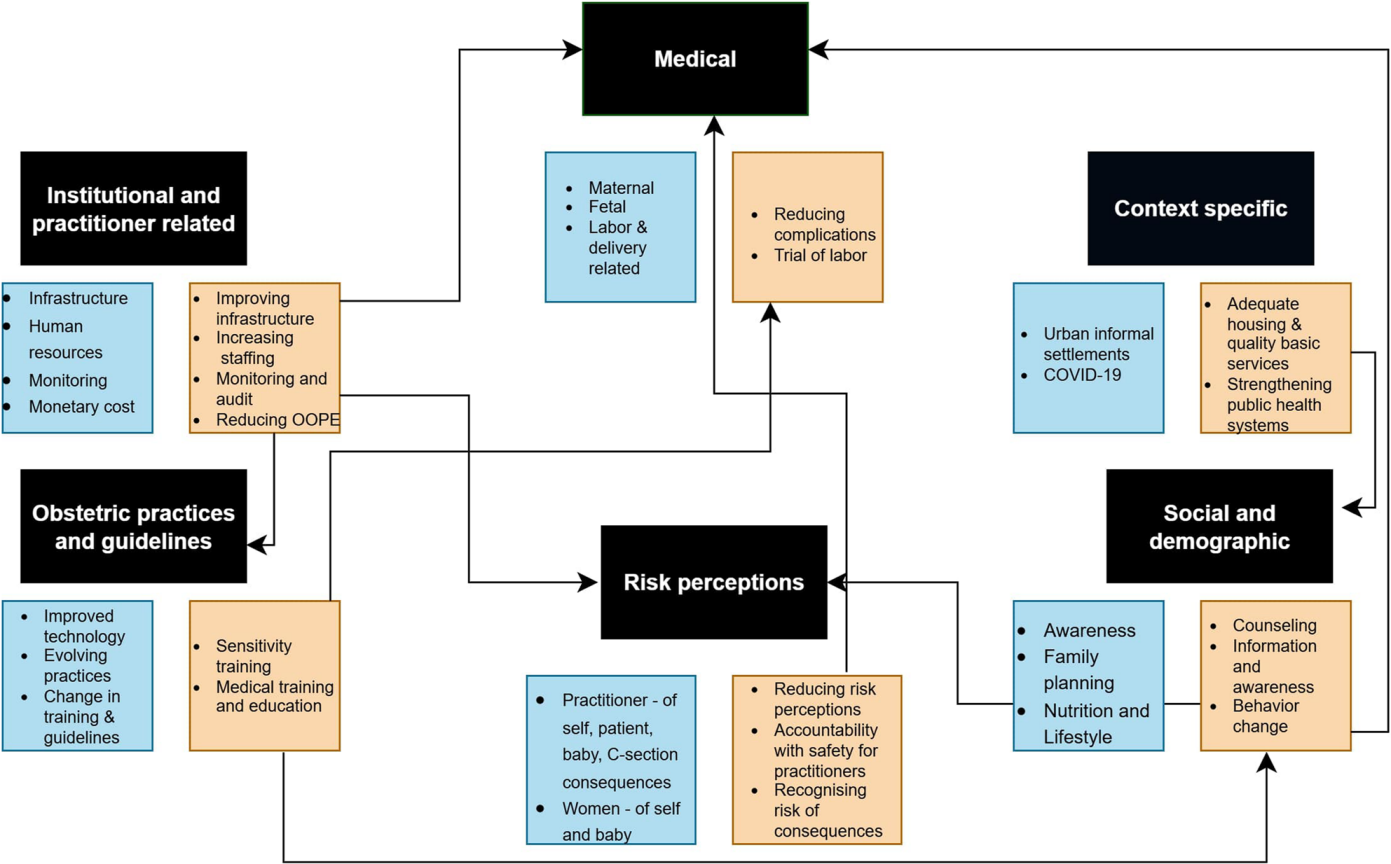
Interconnected reasons for C-sections and corresponding recommendations. Note- OOPE: Out-of-pocket expenditure. Black boxes represent main themes and blue represent sub-themes of identified reasons for C-section; orange boxes represent corresponding recommendations. Arrows represent interconnections between each theme

Figure 2 presents the detailed reasons for C-sections in our context, while providing corresponding measures to address them. The black boxes represent the main themes identified as the reasons for C-sections, while the blue boxes represent the key sub-themes within each. The orange boxes present the recommended measures to address the reasons contributing to C-sections within each theme. The arrows represent the interconnected nature of how one set of recommendations will have an influence on other reasons as well. For instance, improving infrastructure and resources can reduce risk perception among practitioners, reflected in their medical practice, e.g., giving a trial of labor rather than directly preferring a C-section. Similarly, the Figure presents other suggestions and their interconnected impacts, which could collectively address the issues surrounding C-sections and their rise in our setting.

For this, a combination of community-based and systemic interventions is needed to enable better patient-practitioner relations, mitigate adverse outcomes associated with C-sections and reduce avoidable C-sections. While clinical interventions to reduce C-sections are useful [45–47], WHO reports that addressing C-sections through non-clinical interventions could be beneficial in reducing C-Sections [48]. Our study also showed that several non-clinical reasons influenced medical indications for C-section. We therefore focus on addressing key non-clinical reasons to reduce avoidable C-sections. These include both suggestions that can be promptly implemented and wider systemic policy alterations in public health provisioning.

First, we consider that community-based interventions such as counseling by community health workers and non-profits can improve obstetric outcomes in vulnerable communities such as urban informal settlements. Existing community health workers who make regular visits for ANC and PNC services in urban informal settlements need to be trained on special modules related to C-section counselling. Such ANC counselling suggestions can include information on nutrition and physical exercise practices, C-section surgeries and associated myths, and complications during pregnancy and the need to timely address them. Other studies in similar contexts have reported benefits of improved counseling in the ANC period [49]. Similarly, PNC counseling should include the need to continue supplementation, breastfeeding posture, timely check-ups for suture healing, postpartum mental health counseling and adoption of family planning measures. Such interventions can help improve awareness, enable a healthy lifestyle and increase agency, thereby reducing risk perceptions among women and medical complications leading to C-sections.

Secondly, sensitivity training for healthcare staff [46] is required to bridge the gap between patient and practitioner perceptions and to create a collaborative environment of shared agency, ensuring not just access to healthcare but also quality, dignity and respect. For instance, sensitivity towards recognizing women’s risk perceptions of adverse consequences of C-sections can help better address them and also dissipate misconceptions, improving patient-practitioner relations.

Third, there is a need to increase systemic accountability to reduce avoidable C-sections. For this, audit of case-wise medical indications needs to be done in both public and private facilities, monitored by the respective health departments. Some practitioners themselves felt the need for improving monitoring and audit of C-sections; other interventions in LMIC contexts also similarly highlight the need for improved accountability [49]. Along with improved accountability, there is a need to ensure better safety provisions for doctors to enable them to take appropriate clinical decisions.

Finally, recognizing that facilities and practitioners face institutional limitations, there is a need to strengthen health infrastructure and services. Particularly, given that public facilities are crucial health access points for residents in vulnerable settings, we strongly believe that there is a need for state spending on improving staffing and infrastructure of public facilities, also suggested by others [11, 50]. For reducing avoidable C-sections, there is a need for improved training of staff nurses and midwives, use of partographs for monitoring progress of labor, and dedicated staff for monitoring cases with complications where trial of labor can be given. Maternity homes, which act as the closest point of obstetric care for women, need to be strengthened with improved infrastructure and staff. For private facilities, stricter monitoring of licenses to ensure that such facilities meet required infrastructure and staffing norms to provide a safe experience to patients is necessary. Such interventions can help address institutional reasons for C-sections, which also influence doctors’ attitudes, risk perception and decision-making for C-sections. Along with improving health infrastructure, systemic interventions such as improving basic service provisions in urban informal settlements can help ameliorate context-specific issues.

### Limitations

While the study provides valuable insights into the trends and reasons for C-sections, it does face some limitations. The quantitative surveys were cross-sectional; while C-section trends over the years have been presented, causal relationships between independent variables and C-sections cannot be inferred. Additionally, as the analysis pools data from multiple survey rounds conducted across different time points and implementation areas, there may be underlying variations in context that could influence comparability across rounds. The quantitative analysis also had limited variables and didn’t account for other factors that might influence C-section rates, like medical indications and maternal health conditions. The selection of women participants was based on residence within SNEHA’s intervention areas; we acknowledge that insights from women beyond these could have been different. We acknowledge that the research would be enriched by perspectives of other actors such as family members of pregnant women and nurses in health facilities; however, this was beyond the scope of our study. We acknowledge that there may have been a recall bias among participants of the qualitative study, particularly while mentioning their prior experiences, leading to some information gaps. Public practitioners were approached through SNEHA’s prior institutional relations; while this facilitated access, it may have also shaped their responses.

## Conclusion

In conclusion, using a mixed methods approach, our study provides a holistic analysis of a rise in C-sections and the reasons thereof in vulnerable urban settlements in the MMR, India. The study highlights that while C-section rates are rising in urban informal settlements, several reasons such as institutional limitations, evolving obstetric practices, risk perceptions, awareness and lifestyle changes are contributing to it. It recognizes the intertwined nature of such reasons, highlighting the need for a multi-pronged approach with community-based and systemic interventions to reduce avoidable C-sections. A dual perspective, of challenges faced by practitioners and personal experiences and implications of C-section for women, provides critical evidence for informing policy that seeks to optimize obstetric care delivery while balancing safety and patient autonomy. By focusing on the reasons for C-section among women living in vulnerable contexts, accessing primarily public health services, our study provides crucial insights for possible interventions in similar contexts in LMICs to improve patient-practitioner relations and delivery experiences, while bettering obstetric outcomes. 

## Supplementary Information

Supplementary Material 1.

Supplementary Material 2.

## Data Availability

The datasets used and/or analyzed during the current study are available from the corresponding author on reasonable request.

## Abbreviations

ANC: Antenatal care
AOR: Adjusted Odds Ratio
CI: Confidence Interval
CP: Cerebral Palsy
C-section: Cesarean section
IUGR: Intrauterine growth retardation
IVF: In Vitro Fertilization
LMIC: Low-and middle-income countries
MCHN: Maternal and child health and nutrition
MMR: Mumbai Metropolitan Region
NCD: Non-communicable disease
NFHS: National Family Health Survey
NICU: Neonatal Intensive Care Unit
OOPE: Out of pocket expenditure
PIH: Pregnancy-induced hypertension
PNC: Postnatal care
SNEHA: Society for Nutrition, Education and Health Action
TOLAC: Trial of Labor after a previous C-section
VBAC: Vaginal Birth after a previous C-section
WHO: World Health Organization
