# Supplementary material for: “Everywhere you see, C-sections are happening”: examining trends and reasons for a rise in cesarean-section deliveries among women in urban informal settlements, Mumbai Metropolitan Region, using a mixed methods approach
Source: BMC Pregnancy Childbirth. 2026 May 4;26:673. doi: 10.1186/s12884-026-09160-8 (PMC13285392; doi:10.1186/s12884-026-09160-8)
Supplement: Supplementary file 1 — Supplementary Material 1. [file 12884_2026_9160_MOESM1_ESM.docx]

**Annexure 1**

**Quantitative survey questionnaire**

| [**Demographic information**](https://www.commcarehq.org/a/sc-baseline/apps/view/293ed09dc4430b87b6159152d1b6e440/form/0ad1d951b2d14aa695ee21b1f4660328/source/#form/Infoa) | | |
| --- | --- | --- |
| **Say to respondent: I am going to ask you about the people who live in your house, starting with you**. | | |
| Q – 01 | [Total household members](https://www.commcarehq.org/a/sc-baseline/apps/view/293ed09dc4430b87b6159152d1b6e440/form/0ad1d951b2d14aa695ee21b1f4660328/source/#form/hh_members) | □□ |
| Q – 02 | [Type of Family](https://www.commcarehq.org/a/sc-baseline/apps/view/293ed09dc4430b87b6159152d1b6e440/form/0ad1d951b2d14aa695ee21b1f4660328/source/#form/hhtype) | 🞏 Nuclear  🞏 Joint |
| Q – 03 | How old are you? | □□ |
| Q – 04 | How many years of schooling have you had? | □□ |
| Q – 05 | What is your main occupation? | |
|  | - Does not work or looking for work - Student (school or college) - Job that does not require skills or training - Runs machines in a factory, or driver - Makes products - Agriculture or fishery worker | - Shop, market, hotel or transport worker - Junior white collar - Service sector skilled or technical work - Professional - Senior manager or government official |
| Q – 06 | Does your husband live here? | - Husband lives in household - Husband died - Separated, divorced - Lives elsewhere in India - Lives abroad |
| Q – 07 | How old is he? (If unknown, enter 99) | □□ |
| Q – 08 | How many years of schooling has he had? | □□ |

| Q - 09 | What is his main occupation? | | |
| --- | --- | --- | --- |
|  | - Does not work or looking for work - Student (school or college) - Job that does not require skills or training - Runs machines in a factory, or driver - Makes products - Agriculture or fishery worker | - Shop, market, hotel or transport worker - Junior white collar - Service sector skilled or technical work - Professional - Senior manager or government official | |
| Q - 10 | [What is your religion?](https://www.commcarehq.org/a/sc-baseline/apps/view/293ed09dc4430b87b6159152d1b6e440/form/0ad1d951b2d14aa695ee21b1f4660328/source/#form/respreligion) | | |
|  | - Hindu - Muslim - Christian - Buddhist/Neo Buddhist - Parsi/Zoroastrian | | - Sikhism - Jain - Other - [Specify other](https://www.commcarehq.org/a/sc-baseline/apps/view/293ed09dc4430b87b6159152d1b6e440/form/0ad1d951b2d14aa695ee21b1f4660328/source/#form/religionother)…………………………………………………. |
| Q - 11 | How long has the family been living in Mumbai?  (If less than 1 year, enter 0. If since birth, enter 99) | | □□ |
| Q – 12 | [How long have you been living in Mumbai? Enter number of years. (If <1 year, enter 0. If since birth, enter 99)](https://www.commcarehq.org/a/sc-baseline/apps/view/293ed09dc4430b87b6159152d1b6e440/form/0ad1d951b2d14aa695ee21b1f4660328/source/#form/mumdur) | | □□ |
| Q – 13 | [How long have you been living in this](https://www.commcarehq.org/a/sc-baseline/apps/view/293ed09dc4430b87b6159152d1b6e440/form/0ad1d951b2d14aa695ee21b1f4660328/source/#form/areadur) *[basti](https://www.commcarehq.org/a/sc-baseline/apps/view/293ed09dc4430b87b6159152d1b6e440/form/0ad1d951b2d14aa695ee21b1f4660328/source/#form/areadur)*[? Enter number of years](https://www.commcarehq.org/a/sc-baseline/apps/view/293ed09dc4430b87b6159152d1b6e440/form/0ad1d951b2d14aa695ee21b1f4660328/source/#form/areadur)  [(If <1 year, enter in 0. If since birth, enter 99)](https://www.commcarehq.org/a/sc-baseline/apps/view/293ed09dc4430b87b6159152d1b6e440/form/0ad1d951b2d14aa695ee21b1f4660328/source/#form/areadur) | | □□ |
| Q – 14 | In the last one year, have you stayed away from this *basti* for more than one month? required | | - Yes - No |
| Q – 15 | If yes, for how long? Required | | □□ |
| Q – 16 | Does your family own or rent your home? | | - Own - Rent |
| Q – 17 | [Do you own any of the following household items?](https://www.commcarehq.org/a/sc-baseline/apps/view/293ed09dc4430b87b6159152d1b6e440/form/0ad1d951b2d14aa695ee21b1f4660328/source/#form/asset) | | - Mattress - Pressure cooker - Table - Fridge - Color TV - Two-wheeler (motor cycle, scooter) - AC/Cooler - washing machine - Sewing machine |
| Q –18 | [What fuel do you use for cooking?](https://www.commcarehq.org/a/sc-baseline/apps/view/293ed09dc4430b87b6159152d1b6e440/form/0ad1d951b2d14aa695ee21b1f4660328/source/#form/fuel) | | - Wood, charcoal, dung - Kerosene, LPG - LPG - Electricity - Does not cook at home |
| Q- 19 | Interviewer: select the type of roof in the home | | - Concrete, brick, cement (RCC, RBC, cement, concrete) - others |

| Q –20 | [What is the main source of drinking-water for members of your household?](https://www.commcarehq.org/a/sc-baseline/apps/view/293ed09dc4430b87b6159152d1b6e440/form/0ad1d951b2d14aa695ee21b1f4660328/source/#form/dwater) | | |
| --- | --- | --- | --- |
|  | - Piped water into dwelling - Piped water into yard/plot - Piped to neighbors - Public tap/stand pipe - Tubewell or borehole - protected well - unprotected well | - Rainwater - Tanker truck - Cart with small tank - bottled water - Community RO plant - Other - Other specify………………………………………….………. | |
| Q – 21 | What kind of toilet facility do members of your household usually use? | | - Public flush/pour flush toilet (unimproved) - Private/inside home flush toilet (improved) - No facilities or field or road (unimproved) |

| **Maternal history** | | |
| --- | --- | --- |
| Q – 22 | [How many times did you get pregnant: [Total Pregnancy]](https://www.commcarehq.org/a/sc-baseline/apps/view/5518731be73f08ba4ed8ebe77efcc7e2/form/561258af5cb94014bf4b7b11d66112ca/source/#form/total_gravida) | □□ |
| Q – 23 | [How many children do you have?](https://www.commcarehq.org/a/sc-baseline/apps/view/5518731be73f08ba4ed8ebe77efcc7e2/form/561258af5cb94014bf4b7b11d66112ca/source/#form/livebirth) | □□ |
| Q – 24 | [How many times did you get pregnant in the last 5 years?](https://www.commcarehq.org/a/sc-baseline/apps/view/5518731be73f08ba4ed8ebe77efcc7e2/form/561258af5cb94014bf4b7b11d66112ca/source/#form/last5yr_gravida) | □□ |

| [**Gravida_outcome**](https://www.commcarehq.org/a/sc-baseline/apps/view/5518731be73f08ba4ed8ebe77efcc7e2/form/561258af5cb94014bf4b7b11d66112ca/source/#form/gravida_outcome) | | |
| --- | --- | --- |
| Q - 25 | F[irst pregnancy](https://www.commcarehq.org/a/sc-baseline/apps/view/5518731be73f08ba4ed8ebe77efcc7e2/form/561258af5cb94014bf4b7b11d66112ca/source/#form/first_outcome) | - Live birth - Still birth - abortion - Miscarriage |
| Q - 26 | [First Delivery date](https://www.commcarehq.org/a/sc-baseline/apps/view/5518731be73f08ba4ed8ebe77efcc7e2/form/561258af5cb94014bf4b7b11d66112ca/source/#form/first_outcomedate) | ……./……../…………… |
| Q - 27 | [Second pregnancy](https://www.commcarehq.org/a/sc-baseline/apps/view/5518731be73f08ba4ed8ebe77efcc7e2/form/561258af5cb94014bf4b7b11d66112ca/source/#form/second_outcome) | - Live birth - Still birth - Abortion - Miscarriage |
| Q - 28 | [Second Delivery date](https://www.commcarehq.org/a/sc-baseline/apps/view/5518731be73f08ba4ed8ebe77efcc7e2/form/561258af5cb94014bf4b7b11d66112ca/source/#form/second_outcomedate) | ……./……../…………… |
| Q - 29 | [Third pregnancy](https://www.commcarehq.org/a/sc-baseline/apps/view/5518731be73f08ba4ed8ebe77efcc7e2/form/561258af5cb94014bf4b7b11d66112ca/source/#form/third_outcome) | - Live birth - Still birth - Abortion - Miscarriage |
| Q - 30 | [Third Delivery date](https://www.commcarehq.org/a/sc-baseline/apps/view/5518731be73f08ba4ed8ebe77efcc7e2/form/561258af5cb94014bf4b7b11d66112ca/source/#form/third_outcomedate) | ……./……../…………… |
| Q - 31 | [Forth pregnancy](https://www.commcarehq.org/a/sc-baseline/apps/view/5518731be73f08ba4ed8ebe77efcc7e2/form/561258af5cb94014bf4b7b11d66112ca/source/#form/forth_outcome) | - Live birth - Still birth - Abortion - Miscarriage |
| Q - 32 | [Fourth Delivery date](https://www.commcarehq.org/a/sc-baseline/apps/view/5518731be73f08ba4ed8ebe77efcc7e2/form/561258af5cb94014bf4b7b11d66112ca/source/#form/forth_outcomedate) | ……./……../…………… |
| Q - 33 | [Fifth pregnancy](https://www.commcarehq.org/a/sc-baseline/apps/view/5518731be73f08ba4ed8ebe77efcc7e2/form/561258af5cb94014bf4b7b11d66112ca/source/#form/fifth_outcome) | - Live birth - Still birth - Abortion - Miscarriage |
| Q - 34 | [Fifth Delivery date](https://www.commcarehq.org/a/sc-baseline/apps/view/5518731be73f08ba4ed8ebe77efcc7e2/form/561258af5cb94014bf4b7b11d66112ca/source/#form/fifth_outcomedate) | ……./……../…………… |
| Q - 35 | [Sixth [pregnancy](https://www.commcarehq.org/a/sc-baseline/apps/view/5518731be73f08ba4ed8ebe77efcc7e2/form/561258af5cb94014bf4b7b11d66112ca/source/#form/fifth_outcome)](https://www.commcarehq.org/a/sc-baseline/apps/view/5518731be73f08ba4ed8ebe77efcc7e2/form/561258af5cb94014bf4b7b11d66112ca/source/#form/sixth_outcomedate) | - Live birth - Still birth - Abortion - Miscarriage |
| Q - 36 | [Sixth Delivery date](https://www.commcarehq.org/a/sc-baseline/apps/view/5518731be73f08ba4ed8ebe77efcc7e2/form/561258af5cb94014bf4b7b11d66112ca/source/#form/sixth_outcomedate) | ……./……../…………… |

| [**Child Under two years**](https://www.commcarehq.org/a/sc-baseline/apps/view/5518731be73f08ba4ed8ebe77efcc7e2/form/561258af5cb94014bf4b7b11d66112ca/source/#form/Maternal_Care) | | |
| --- | --- | --- |
| Q - 37 | Enter the date of birth (When did you have your youngest (surviving) child?) | ………..…./….……/………………(DDMMYYYY) |
| Q - 38 | Enter the sex of the child | - Male - Female |
| **0-2 years (Antenatal care)** | | |
| Q - 39 | Did you receive any antenatal care when you were pregnant with (NAME)? | 🞏 Yes  🞏 No |
| Q - 40 | Where did you go for ANC visits? | - Municipal Health Post - Municipal Maternity hospital - Municipal Hospital - Private facility - Govt. Hospital - Trust Hospital - Field camp - Other |
| Q - 41 | When was the pregnancy registered? | □□ |
| Q - 42 | How many times did you receive antenatal care during last pregnancy? | □□ |
| Q - 43 | Where did you receive most of the antenatal care for this pregnancy? | - Govt. Hospital/BMC hospital - BMC Health Post - BMC Outreach camps - NGO - Private Hospital/clinic - Availed outside the Mumbai (e.g., native place) - Not availed - Other |
| Q - 44 | How many times did you received tetanus (TT/Buster) injection? | □□ |
| Q - 45 | During your last pregnancy did you receive folic acid in first trimester? | - Yes - No |
| Q - 46 | During your last pregnancy, did you consume following tablets/syrups? | - Iron Folic Acid (IFA) tablets - calcium - Nothing |
| Q – 47 | Was the baby born in Mumbai or outside? | - Mumbai - outside Mumbai |
| Q - 48 | Was the baby born in a health facility or at home? | - Facility - Home |
| Q – 49 | At which facility did the delivery happen? | - Municipal health post - Municipal Maternity hospital - Municipal Hospital - Private facility - Govt. Hospital - Trust Hospital - Other |
| Q – 50 | Which type of delivery you had? | - Normal/vaginal - LSCS |
| Q – 51 | Enter the birth weight of the child  (e.g. 2500, If not, then write 9999) | □□□□ |
| Q – 52 | After your discharge, have you gone to see a doctor? | - No - Within 2 days of childbirth - Between 3 – 7 days after childbirth - Between 8 – 42 days after childbirth - Stayed in hospital for long time after childbirth |
