# Supplementary material for: “Everywhere you see, C-sections are happening”: examining trends and reasons for a rise in cesarean-section deliveries among women in urban informal settlements, Mumbai Metropolitan Region, using a mixed methods approach
Source: BMC Pregnancy Childbirth. 2026 May 4;26:673. doi: 10.1186/s12884-026-09160-8 (PMC13285392; doi:10.1186/s12884-026-09160-8)
Supplement: Supplementary file 2 — Supplementary Material 2. [file 12884_2026_9160_MOESM2_ESM.docx]

Annexure 2

Qualitative Interview Guide

| **Women who have had C-section Deliveries**  *Give SNEHA’s introduction, introduction about the research and information needed* | |
| --- | --- |
| Demographic Details | Name  Contact  Age  Highest Educational Qualification Occupation  Years of residence  Gravida Count Number of children |
| Basic Details about C- section deliveries | How many C-section deliveries did you have? (based on gravida) When did you have your delivery? (note month and year for each)  Which health facility did you access (public/private; hospital/maternity home/clinic etc.) and why? (Record name of the facility as well)  Was there a change in the facility during your pregnancy, why? What information did you have regarding C-section surgeries before your pregnancy?  Do you think it is preferable to have vaginal delivery or C-  section? |
| Pre-  delivery/Pregnancy Details related to Awareness and Complications | Did you register for public Ante-Natal Care? How often did you visit?  Did you register at the neighbourhood Anganwadi or with CHWs during your pregnancy?  What was your interaction with CHW and/or SNEHA volunteers/staff during and after your pregnancy?  Were you informed about C-section during your pregnancy? By whom? What was the information given to you? (Probe- given as an option/choice, conditions where C-section necessary known, adverse impacts and complications known)  How was your health during the pregnancy period? Did you face any complications? How did you deal with them/what was suggested by health provider?  What were the precautions that you had taken during your pregnancy for a healthy delivery? (Probe- nutrition, supplements, exercise, etc.) What were the suggestions in this regard from the health provider?  How often did you get your vitals checked during pregnancy?  Where? (Probe- high blood pressure, blood sugar, sonography) |
| Experience of C- section Delivery- understanding agency and reasons reported | Can you describe the events leading to your C-section?  Was your surgery planned or decided beforehand or was it an emergency?  If elective- Did you decide that you wanted a C-section before your delivery date? Why? In consultation with whom?  If planned- Who suggested having a C-section? What were the reasons given? Were there any complications? How did you decide? (Probe- were you unsure, who did you consult, who finally decided, did you feel pressurized into doing a C-section, by whom)  If emergency- Who suggested having a C-section? What were  the reasons given? (Probe- what were the complications, was it  before you went into labour or during labour) How did you decide? (Probe- were you unsure, who did you consult, who decided finally, did you feel pressurized into doing a C-section, by whom)  What was your experience during the surgery? (Probe- do you feel the staff was polite, co-operative, did they explain you the details of the surgery, possible complications/side effects, etc., did they provide adequate attention and care immediately after the surgery?)  What was the total cost incurred? |

| Post-delivery challenges for maternal and newborn health | For how many days were you in the hospital after the surgery? Was your newborn with you at that time? If not- reasons  Did the staff inform you about starting breastfeeding within an hour, exclusive breastfeeding for two days, etc.?  Did you do any post-surgery follow-ups? Was it at the same place as delivery? How was your experience?  It has been (state the duration) months since your delivery. Can you tell me any challenges or issues you have faced regarding – your physical health, specific complications (example high BP if continued), nutrition, mental health, weakness etc.  Can you tell me any issues you have faced regarding your mental and emotional health after the surgery?  Did you face any issues with respect to childcare such as breastfeeding, overall health of the child?  How did you address these issues?  Did you receive adequate support from your family and husband post-surgery? |
| --- | --- |
| Relation of C-section to COVID-19 | Did you have any deliveries /pregnancy during the COVID-19 pandemic?  If yes- What were the specific challenges that you faced due to the pandemic during your pregnancy (Probe- access to healthcare, complications in pregnancy, decision regarding C- section, difficulties during delivery, difficulties after delivery) Was the decision for C-section taken due to COVID or other  related factors? |
| Overall perceptions  /preferences about C- section and its reasons | According to you, why do you think you had to go through C- section instead of a normal delivery?  If has had a normal delivery before- how do you think was your overall experience with the health facilities different – if you had a normal delivery before what do you think was different this time? Would you prefer C-section or normal delivery in case of a future pregnancy? What precautions would you take for it?  Have you heard of other women in the neighbourhood who had C-sections- have they had similar experiences as yours (Probe if they know of a case)  Why do you think C-sections are rising in the area and in the city? |

| **Health Obstetric Staff**  *Give SNEHA’s introduction and partnerships with health systems, introduction about the research and information needed* | |
| --- | --- |
| Demographic Details | Name  Contact  Age Gender  Years of Experience  Details of Experience- Degree, which hospitals/facilities have worked in |
| General Perspective about deliveries and reasons for rise in C-sections | Do you think C-section deliveries have increased? Among whom? (Probe- socio demographics) We have seen that there is a rise in C-section deliveries in the city over the years. What based on your experience could be the reasons for the same?  According to you, do women from (name the area) use your health facility for delivery?  Why do you think women from (name the area) use public/private facilities for delivery (ask based on public or private facility)? (Probe- those mentioned by women- cost, overburdened staff, unavailability of male doctors, sterilization)  We have seen an increase in C-section deliveries among women in (name the area). What do you think could be the reasons for the same? |
| Exploring reasons for C-section | What are the situations/medical and non-medical indications based on which you suggest a C-section? (Probe the criteria mentioned by women that we have recorded- water break but labour not induced, labour not getting induced even after giving injections; after due date, twin birth, child defecated in the womb, narrow uterine cavity, cervix not dilated, breech position of baby, amniotic fluid was less, high/low BP, umbilical cord around neck of baby, prior C-section, high heartrate of baby.)  Do you think C-sections have risen due to complications? What are the most prevalent complications based on your experience? Why do you think such complications have increased/take place? (Probe from those mentioned by women participants)  Do you/how do you think can these be avoided?  In the case of previous C-section, is a C-section necessarily recommended? What are the conditions or not for the same?  Have you ever suggested against a C-section?  Among elective, planned and emergency C-sections which are the most commonly encountered by you and why?  (Probe-According to you are C-sections most commonly decided on day of delivery or during delivery process than beforehand and why)  In cases where patients have asked for C-section delivery what are the most common reasons? What is your response in such cases?  In what conditions do you ask for a planned surgery to be done?  In what situations (that are neither elective nor planned) do you first try for normal delivery and then suggest C-section and in what cases do you directly suggest C-section and why?  (Probe if they can narrate a case regarding each of these- emergency, planned, elective)  Do you think there are cases of avoidable C-sections taking place  in the city? Why?  In cases where a woman has more than two children do you necessarily suggest sterilization along with the C-section surgery? (Probe- reasons for suggesting sterilization like government rules, biases etc., ask whether patients/families themselves suggest) |
| Agency in decisions regarding C-sections | When a C-section has to be done who do you approach for consent? In most cases who is the decision maker regarding whether a C-section has to be done or not? Do you inform the pregnant woman regarding C-section, is her consent mandatory? What is the information that you provide to the woman/family regarding the surgery? Do you inform about the pros and cons if  any? What are the consent forms to be signed by the patient/family?  What is the most common response that you receive when you tell patients/family about having to do a C-section? |
| ANC | When do most women register for ANC care and do they visit regularly? Is the ANC staff different from those doing delivery/C-section?  What information regarding C-section and about a possibility of C-section during their delivery is given to pregnant women who have registered for ANC? |

| Post-operative care | What is the post-surgery care followed by the facility?  Do you ensure that the newborn is able to breastfeed within one hour? In what conditions does this become less possible?  What according to you could be the potential risks and downsides of a C-section for women and their newborn?  Have you experienced women who have had C-section visit you regarding any complications or health concerns post-surgery?  Provide details |
| --- | --- |
| Institutional Challenges | Does your facility have availability of doctors/infrastructure for conducting C-sections 24x7? (Probe for particular timings, unavailability of doctors, transferring patients, having only planned surgeries)  In what cases of delivery complications do you transfer a patient to another hospital?  What are the differences in the challenges faced as a doctor in normal vs C-section deliveries? (Probe for- staff shortage, time, overburdened, skill etc.)  Do you have rules prescribed by your institution/management regarding when C-section deliveries have to be done or a preference related to C- section? (Probe- incentives if any associated with surgeries performed, monitoring of practitioner-wise C-section deliveries to total)  What were the challenges faced for deliveries during the COVID-  19 pandemic? Was there an increased need for C-section deliveries during that time? Why? |
